# Mitofusin 2 controls mitochondrial and synaptic dynamics of suprachiasmatic VIP neurons and related circadian rhythms

**DOI:** 10.1172/JCI185000

**Published:** 2025-07-01

**Authors:** Milan Stoiljkovic, Jae Eun Song, Hee-kyung Hong, Heiko Endle, Luis Varela, Jonatas Catarino, Xiao-Bing Gao, Zong-Wu Liu, Peter Sotonyi, Sabrina Diano, Jonathan Cedernaes, Joseph Bass, Tamas L. Horvath

**Affiliations:** 1 Department of Comparative Medicine, Yale University School of Medicine, New Haven, Connecticut, USA.; 2 Department of Pharmacology, University of Nis School of Medicine, Nis, Serbia.; 3 Department of Medicine, Division of Endocrinology, Metabolism and Molecular Medicine, Northwestern University Feinberg School of Medicine, Chicago, Illinois, USA.; 4 Department of Molecular and Translational Neuroscience of Anatomy II, University of Cologne, Cologne, Germany.; 5 Universidad da Coruña, FeMCa, CICA – Centro Interdisciplinar de Química e Bioloxía, Rúa As Carballeiras, A Coruña, Spain, and Universidade da Coruña, MEtCaEn, Departamento de Fisioterapia, Médicina e Ciencias Biomédicas, Facultade de Ciencias da Saúde, A Coruña, Spain.; 6 Department of Anatomy and Histology, University of Veterinary Medicine, Budapest, Hungary.; 7 Department of Physiology and Institute of Human Nutrition, Columbia University Irving Medical Center, New York, New York, USA.

**Keywords:** Cell biology, Metabolism, Neuroscience, Behavior, Mitochondria, Synapses

## Abstract

Sustaining the strong rhythmic interactions between cellular adaptations and environmental cues has been posited as essential for preserving the physiological and behavioral alignment of an organism to the proper phase of the daily light/dark (LD) cycle. Here, we demonstrate that mitochondria and synaptic input organization of suprachiasmatic (SCN) vasoactive intestinal peptide–expressing (VIP-expressing) neurons showed circadian rhythmicity. Perturbed mitochondrial dynamics achieved by conditional ablation of the fusogenic protein mitofusin 2 (Mfn2) in VIP neurons caused disrupted circadian oscillation in mitochondria and synapses in SCN VIP neurons, leading to desynchronization of entrainment to the LD cycle in Mfn2-deficient mice that resulted in an advanced phase angle of their locomotor activity onset, alterations in core body temperature, and sleep-wake amount and architecture. Our data provide direct evidence of circadian SCN clock machinery dependence on high-performance, Mfn2-regulated mitochondrial dynamics in VIP neurons for maintaining the coherence in daily biological rhythms of the mammalian organism.

## Abstract 2

This study showed that mitochondrial dynamics governed by the molecule, mitofusin 2, controls synaptic plasticity of neurons of the suprachiasmatic nucleus of the hypothalamus and their functional output.

## Introduction

The circadian rhythm is an endogenous oscillation in behavioral, physiological, and other bodily functions has a periodicity of approximately 24 hours. The hypothalamic suprachiasmatic nucleus (SCN) is the master circadian pacemaker, which, by transcriptional/translational negative feedback loop–dependent gene expression and intrinsic neuronal circuitry, entrains other brain regions and peripheral tissues to synchronize their activities in coherence with environmental time cues (1, 2). The SCN directly receives light input from the photosensitive retinal ganglion cells via the retinohypothalamic tract, which heavily terminates into its ventral subdivision (SCN core) mainly containing vasoactive intestinal peptide–expressing (VIP-expressing) neurons (2). These neurons densely project outside the SCN core, influencing other neuronal cell populations in its dorsal subdivision (SCN shell) that express the VIP VPAC2 cognate receptors. This anatomical feature reflects the vast influence that VIP neurons have on the entire SCN circuit synchronization with the light/dark (LD) cycle and ultimately in sustaining circadian rhythms at the molecular, cellular, and behavioral levels (3–5). Accordingly, previous studies showed that developmental disruption of the VIP neurons or VPAC2 receptors can severely compromise both the synchrony in the SCN neuronal network and circadian amplitude (6–8), whereas targeted optogenetic activation of VIP neurons can adequately reset the circadian phase of the SCN ensemble (9).

The daily fluctuation of SCN gene expression and neuronal firing requires precise rhythmic regulation of local metabolic activity to meet its bioenergetic demand. Indeed, a rhythmic pattern in the expression levels of several genes involved in the control of metabolism was observed within the SCN (10, 11). Furthermore, it was suggested that SCN metabolic rhythmicity is intertwined with mitochondrial morphological and functional plasticity (12–14). Ample evidence showed that in fulfilling cell-specific bioenergetic demand, mitochondria undergo tightly coordinated dynamic morphological changes reflected in their continuous fusion and fission (15, 16). Previous studies (17, 18) suggested the relationship between this mitochondrial adaptation and the circadian phase in the peripheral tissues, however, data directly connecting SCN clock machinery function with mitochondrial dynamics therein are scarce. Given the indispensable role of SCN VIP neurons in maintaining circadian rhythm (5), we examined here the effect of mitofusin 2 (Mfn2), a mitochondrial membrane protein involved in the control of the fusion process (19), on SCN VIP neuronal activity and downstream effects on behavior, thermoregulation, and sleep.

## Results

### Daily remodeling of synapses and mitochondria in the SCN VIP neurons is diminished in constant dark.

**Figure 1:**
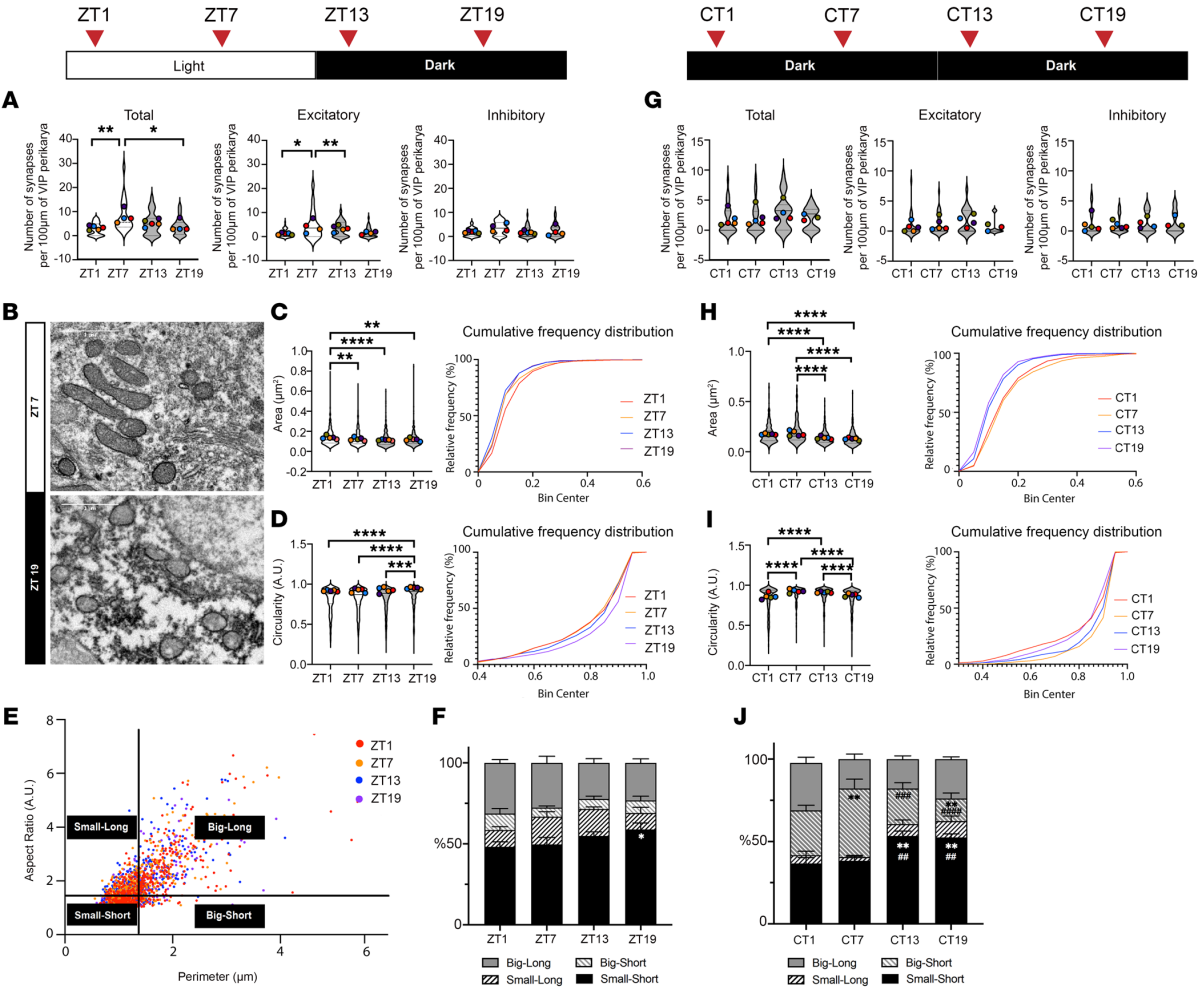
Circadian rhythm of synaptic innervation and mitochondrial morphology of SCN VIP neurons in LD and DD environments. (**A**–**F**) Analyses of C57BL/6J mice housed in LD conditions (ZT0: lights on; ZT12: lights off). (**A**) Synapse density of SCN VIP-immunolabeled neurons measured using an electron microscope at ZT1, ZT7, ZT13, ZT19 under a normal LD cycle. (**B**) Representative electron microscopic images of mitochondria in SCN VIP neurons at ZT7 and ZT19. Scale bar: 1 μm. (**C**) Cross-sectional area and (**D**) circularity of mitochondria in SCN VIP neurons and their cumulative probability distributions at ZT1, ZT7, ZT13, and ZT19 in LD. (**E**) Aspect ratio and perimeter of mitochondria in SCN VIP neurons. The average value of the aspect ratio was used to determine the long/short group, and the average value of perimeter was used to determine the big/small group. (**F**) Percentage of 4 different groups of mitochondria in SCN VIP neurons at ZT1, ZT7, ZT13, and ZT19 under LD conditions. Small/short group at ZT1 versus ZT19; *P = 0.0372. (**G**–**J**) Analyses of C57BL/6J mice released into DD for 48 hours. (**G**) Density of synapses on SCN VIP neurons measured using an electron microscope at CT1, CT7, CT13, CT19 (DD condition). (**H**) Cross-sectional area and (**I**) circularity of mitochondria in SCN VIP neurons and their cumulative probability distributions at CT1, CT7, CT13, CT19 (DD condition). (**J**) Percentage of 4 different groups of mitochondria in SCN VIP neurons at CT1, CT7, CT13, CT19 (DD condition). Small/short group at CT1 versus CT13; **P = 0.013; CT1 versus CT19, **P = 0.015; CT7 versus CT13, ^##^*P* = 0.0069; CT7 versus CT19, ^##^*P* = 0.0086. Big/short group CT1 versus CT7, **P = 0.0072; CT1 versus CT19, **P = 0.0082; CT7 versus CT13, ^###^*P* = 0.0002; CT7 versus CT19 ^####^*P* < 0.0001. Approximately 5 cells per mice; 4–5 mice per time point (see also Supplemental Table 1 for details on the statistical information for each graph). Kruskal-Wallis with Dunn’s test for **A**–**D** and **G**–**I**; 2-way ANOVA with Tukey’s test for **F** and **J**. *P < 0.05, **P < 0.01, ***P < 0.005, and ****P < 0.0001.

To determine the effect of the SCN circadian oscillation in the LD cycle at the cellular level, we first analyzed synapse density and mitochondrial morphology in SCN core VIP- expressing neurons of C57BL/6J mice at Zeitgeber times (ZT) 1, 7, 13, 19, (ZT0: lights on; ZT12: lights off). Our electron microscopy analysis of VIP-immunolabeled SCN neurons revealed significantly higher numbers of synapses and, specifically, excitatory synapses in neuronal somata at ZT7 compared with the other time points measured (Figure 1A). Analysis of the mitochondria in these neurons revealed that they had a larger area and perimeter during the light phase, but that their shape was more circular during the dark phase (Figure 1, B–D, and Supplemental Figure 1, A and B; supplemental material available online with this article; https://doi.org/10.1172/JCI185000DS1). To better characterize changes in mitochondrial morphology, we established criteria based on measures of the perimeter (size) and aspect ratio (shape) of an individual mitochondrion. Then, we classified mitochondria in SCN VIP neurons into “big” and “small” groups and “long” and “short” groups (Figure 1E) and found an inverse relationship between the “small-short” group and the “big-long” group. The ratio of the “big-long” group increased during the light phase and decreased during the dark phase, while it had the opposite trend for the “small-short” group (Figure 1F). Mitochondrial density and coverage of the cytosolic area in SCN VIP neurons showed no change throughout the day (Supplemental Figure 1, C–E).

Next, we repeated the experiment with the mice housed in constant darkness (DD) for 48 hours. The oscillation of synapse density in SCN VIP neurons was diminished with the absence of light signals (Figure 1G), suggesting that the remodeling of circadian synaptic innervation of these neurons was related to photic integration. During DD, we observed an interesting change in the morphology of mitochondria. While the SCN VIP mitochondria size oscillations maintained the same pattern as in the LD condition, their shape drastically changed in the absence of light. The most noticeable change in mitochondrial morphology in the DD condition was the increase of “big-short” mitochondria overall, peaking at the circadian time point CT7 (corresponding to ZT7), in these neurons (Figure 1, H–J, and Supplemental Figure 1, F and G). At circadian time points CT13 and CT19, the number of mitochondria was significantly higher, but we found no change in the mitochondrial cytosolic coverage (Supplemental Figure 1, H and I). These data indicated a shift of mitochondrial dynamics toward fission at ZT13 and ZT19 under DD. While the “small-short” group maintained a similar circadian pattern, the “big-long” group changed its pattern in the DD condition when compared with the LD condition.

### Mfn2 deletion disrupts the daily remodeling of mitochondrial architecture in the SCN VIP neurons.

**Figure 2:**
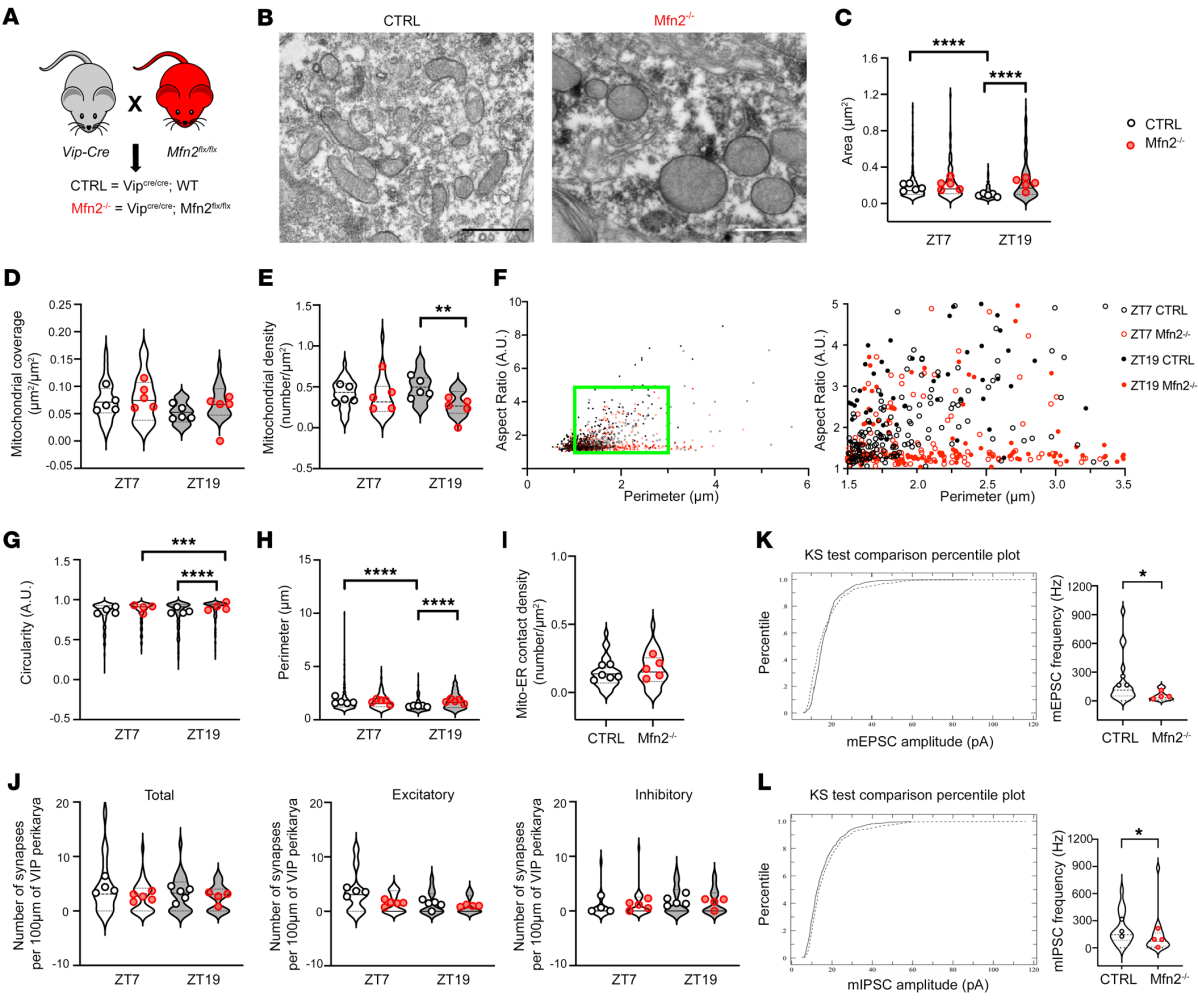
Loss of Mfn2 alters mitochondrial morphology and synaptic innervations of SCN VIP neurons. (**A**) Generation scheme for mice with Mfn-depleted SCN VIP neurons. Mfn2^fl/fl^ mice were crossed with VIP-Cre–transgenic mice to generate VIP^Cre/Cre^ Mfn2^fl/fl^ (Mfn2^–/–^) and control VIP^Cre/Cre^ (CTRL) mice. (**B**) Representative electron microscopic images of mitochondria in VIP neurons in the SCN of control and Mfn2^–/–^ mice at ZT7. Scale bars: 1µm. (**C**) Cross-sectional area of mitochondria, (**D**) mitochondrial coverage, and (**E**) mitochondrial density of cytoplasm in SCN VIP neurons of control and Mfn2^–/–^ mice at ZT7 and ZT19. (**F**) Aspect ratio and perimeter of mitochondria in SCN VIP neurons of control and Mfn2^–/–^ mice. The green box indicates the location of the zoomed-in version of the graph on the right. (**G**) Circularity and (**H**) perimeter at ZT7 and ZT19, (**I**) mitochondria (Mito) and ER contact per SCN VIP neuron of control and Mfn2^–/–^ mice at ZT7, and (**J**) number of synapses in VIP cells at ZT7 and ZT19. *n* = approximately 5 cells per mouse; *n* = 4–6 mice per time point. (**K**) Cumulative distribution of mEPSC amplitude and frequency of SCN VIP neurons in control (*n* = 20) and Mfn2^–/–^ (*n* = 16) mice measured at ZT7. (**L**) Cumulative distribution of mIPSC amplitude and frequency of SCN VIP neurons in control (*n* = 19) and Mfn2^–/–^ (*n* = 20) mice measured at ZT7. Kruskal-Wallis with Dunn’s test for **C**, **E**–**H**, and **J**; 1-way ANOVA with Tukey’s test for **D**; Mann Whitney test for **I**; Kolmogorov-Smirnov test and Mann Whitney test for **K** and **L**. *P < 0.05, **P < 0.01, ***P < 0.005, and ****P < 0.0001. (See also Supplemental Table 1 for details on the statistical information for each graph.)

Mitochondria undergo remodeling through fusion and fission to alter their distribution, morphology, and function in response to changes in their environment. To explore the effect of mitochondrial dynamics in the SCN circadian rhythmicity, we generated mice lacking Mfn2 (Mfn2^–/–^) in VIP neurons (Figure 2A). The loss of Mfn2 resulted in abnormal enlargement and circularity of mitochondria in SCN VIP neurons compared with mitochondria from the control mice in the dark (ZT19) phase (Figure 2, B and C). In the same phase, mitochondrial cytosolic coverage in Mfn2^–/–^ neurons did not change, but the density of mitochondria was reduced, likely due to an increase in their size (Figure 2, D and E). These data indicate that the deletion of Mfn2 disrupted the mitochondrial dynamics in SCN VIP neurons. Unlike mitochondria in control SCN VIP neurons, in which their perimeter and aspect ratio were positively correlated, a large portion of mitochondria in Mfn2^–/–^ neurons had their aspect ratio at 1 regardless of their perimeters (Figure 2F). Also, we found that SCN VIP neurons of Mfn2^–/–^ mice had significantly increased circularity and perimeter during ZT19 (Figure 2, G and H), suggesting a higher number of “big-short” mitochondria similar to that observed in mice housed under the DD condition. In addition, no difference in mitochondria-ER contacts in SCN VIP neurons was observed between the 2 groups of animals (Figure 2I).

### Loss of Mfn2 in VIP neurons alters the synchronization of SCN neuronal activity.

Along with mitochondria modifications in SCN VIP neurons of Mfn2^–/–^ mice, we analyzed daily rearrangements of the number of synapses in these neurons. In control animals, the total number of synapses, and particularly excitatory synapses, in SCN VIP neurons generally followed a similar pattern of reduction during the dark phase (ZT19) compared with the light phase (ZT7), consistent with trends previously observed in C57BL/6J mice, even though no significant differences were found. In contrast, Mfn2^–/–^ mice had nearly the same number of SCN VIP synapses regardless of the diurnal phase (Figure 2J). This indicates impairment of circadian oscillation in SCN innervation in Mfn2^–/–^ mice likely because of deficient photic integration in SCN VIP neurons, which, together with their “big-short” mitochondrial phenotype, implies the involvement of mitochondrial dynamics in the light transduction process of SCN VIP neurons.

**Figure 3:**
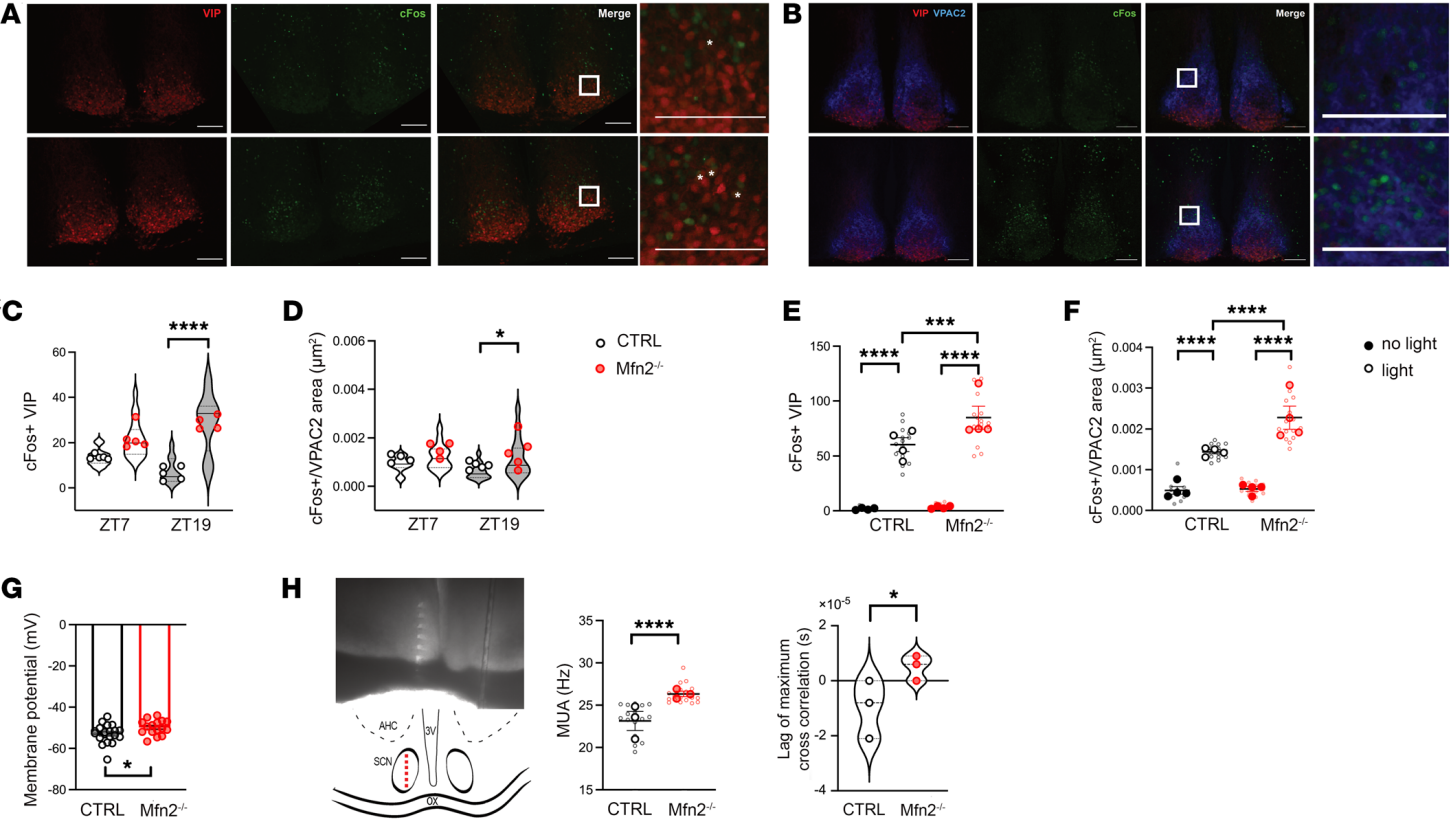
Loss of Mfn2 in VIP neurons alters the synchronization of SCN neuronal activity. (**A** and **C**) Representative confocal images (**A**) of VIP neurons (red) and cFos (green) in the SCNs and (**C**) the number of cFos^+^ SCN VIP neurons in control and Mfn2^–/–^ mice at ZT7 and ZT19. Scale bars: 100 µm; insets 10 µm. (**B** and **D**) Representative confocal images (**B**) of VIP (red), VPAC2 (blue), and cFos (green) expression and (**D**) density of cFos^+^ cells in the VPAC2-expressing area in the SCN of control and Mfn2^–/–^ mice at ZT7 and ZT19. Analysis is of 3 sections per mice (*n* = 4–5 mice/group). Scale bars: 100 μm. (**E**) Number of cFos-expressing SCN VIP neurons and (**F**) density of cFos-expressing cells in the VPAC2-expressing area in the SCN of control and Mfn2^–/–^ mice after exposing to light for 1 hour at ZT13 (light) or stayed in the dark (no light). Analysis is of 4 sections per mice (*n* = 4–5 mice/group). (**G**) Membrane potential of SCN VIP neurons in control (*n* = 20) and Mfn2^–/–^ (*n* = 16) mice. (**H**) Ex vivo MUA recordings across the entire dorsoventral span of SCN and cross-correlation analysis between dorsal and ventral neuronal population rates in control and Mfn2^–/–^ mice (*n* = 3/group). The microphotograph and diagram depict the recording site locations in the SCN (labeling: 3V, third ventricle; OX, optic chiasm; AHC, anterior hypothalamic area central part). Kruskal-Wallis with Dunn’s test for **C**; 1-way ANOVA with Tukey’s test for **D**–**F**; unpaired 2-tailed *t*-test for **G** and **H** (right panel); Mann Whitney test for **H** (left panel). *P < 0.05, ***P < 0.005, and ****P < 0.0001. (See also Supplemental Table 1 for details on the statistical information for each graph.)

To further investigate the changes observed in Mfn2^–/–^ mice, miniature excitatory postsynaptic currents (mEPSCs) and miniature inhibitory postsynaptic currents (mIPSCs) were recorded from SCN VIP neurons using brain slices. We detected a decrease in both mEPSCs and mIPSCs in Mfn2^–/–^ mice compared with controls (Figure 2, K and L) at ZT7, indicating compromised synaptic functions of their VIP neurons. When measuring neuronal activation in the SCN VIP neurons at ZT7 and ZT19 by the analysis of cFos expression, we surprisingly observed significantly higher VIP cFos positivity in Mfn2^–/–^ mice than in control mice at ZT19, despite the findings of synaptic impairments in these animals (Figure 3, A and C). Moreover, at the same time point, we found an increased density of VPAC2 cFos–expressing cells in the SCN nuclei of Mfn2^–/–^ mice (Figure 3, B and D). This aligns with the previous observation (9) of significant cFos positivity induction in the entire SCN after optogenetic activation of VIP neurons. Overall, these findings suggest that the increased SCN VIP activation in Mfn2^–/–^ mice was likely due to a compensatory mechanism potentially arising from Mfn2 loss of function during their developmental period. To clarify this further, future research using conditional-knockout models or tools for temporally controlled gene knockdown will be essential.

Next, we quantified cFos expression in the SCN of Mfn2^–/–^ and control mice exposed to light at ZT13 for 1 hour. We observed increased light-induced activation of SCN VIP- and VPAC2-expressing neurons in Mfn2^–/–^ (Figure 3, E and F). To examine the underlying mechanism of this aberrant neuronal activation, we performed ex vivo electrophysiology recordings in 2 groups of animals to measure the membrane potential of the SCN VIP neurons and found that Mfn2^–/–^ mice had more depolarized membranes of VIP neurons than did their control counterparts (Figure 3G). Also, using a multielectrode array placed across the whole dorsoventral dimension of SCN to record the neuronal spiking activity in the entire structure, we detected significantly (*P* < 0.0001) higher activity in Mfn2^–/–^ mice than in controls. Testing SCN neuronal activity synchronization, we found a difference in the lag between signals corresponding to the peak correlation of the ventral and dorsal neuronal cell population rates in control mice compared with Mfn2^–/–^ mice (Figure 3H), suggesting altered SCN neuronal synchronization in these mice. Although this result implies global dysfunction of their SCN neural circuitry, the role of VIP neurons was probably significant, as evidenced by the higher cFos expression and membrane depolarization. This may be attributed to the unique electrophysiological characteristics of VIP cells, which have a lower firing threshold and an increased spiking probability, as previously described (9, 20). Together, these data indicate that the functional neuronal changes caused by Mfn2 deletion in VIP neurons altered the activity of the entire network, which could, in turn, have altered the synchronization property of the SCN and its response to light stimulation.

### Loss of Mfn2 in VIP neurons disrupts the onset of activity and alters diurnal rhythms.

**Figure 4:**
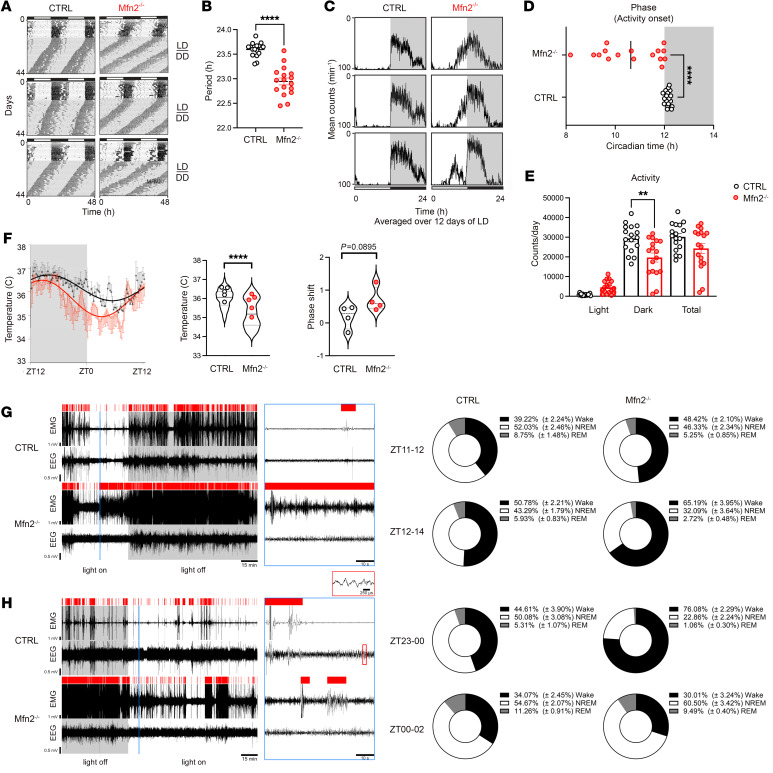
Loss of Mfn2 in VIP neurons alters the diurnal activity rhythm and activity onset. (**A**) Representative actogram showing wheel-running activity of control and Mfn2^–/–^mice. Mice were maintained on a 12-hour light/12-hour dark cycle in wheel cages for 16 days prior to release to DD. (**B**) Free-running endogenous circadian period distributions of control and Mfn2^–/–^ mice. (**C**) Representative activity profile graph of wheel-running activity. The activity profile was averaged over 12 days of LD (days 5–16). (**D**) Phase of activity onset relative to the dark phase and (**E**) distribution of activity counts in the light or dark periods and total activity in control and Mfn2^–/–^ mice averaged for days 5–16 in the LD condition. (**F**) Core body temperature profiles over the entire LD cycle in the control (black) and Mfn2^–/–^ mice (red). (**G** and **H**) Sleep-wake pattern measured during transitions from the light to the dark phase (**G**) and the dark to the light phase (**H**). Unfiltered EEG and EMG signals and distribution of automatically detected vigilant states in 1 control mouse and 1 Mfn2^–/–^ mouse during the last hour of the light or dark phase, and the first 2 hours after shifting in the opposite phase. Panels on the right show extracted signals (framed in blue) in 1-minute time intervals. Red bars over the traces mark wake episodes. Inset in the red rectangle shows the EEG signal with sleep characteristic slow waves. Pie charts show the percentage distribution of wake (black), NREM (white), and REM (gray) episodes in control and Mfn2^–/–^ mice (*n* = 5 for each). ZT23–00: control versus Mfn2^–/–^ mice, *P* = 0.019 for wake; *P* = 0.014 for NREM. Unpaired 2-tailed *t*-test for **B**, **D**, and **F** (right panel); 1-way ANOVA with Tukey’s test for **E**; Mann Whitney test for **F** (left panel); multiple unpaired t-tests with Welch corrections with False Discovery Rate multiple comparisons and 2-stage step-up (Benjamini, Krieger, and Yekutieli) method for **G** and **H**. **P < 0.01, and ****P < 0.0001. (See also Supplemental Table 1 for details on the statistical information for each graph.)

To determine the systemic consequences of Mfn2 deletion–induced alterations in SCN VIP synapses and mitochondria, we measured the circadian rhythm of locomotor activity under normal LD conditions, followed by DD. We found that both Mfn2^–/–^ and control mice had preserved circadian locomotor rhythmicity under DD, but Mfn2^–/–^ mice displayed an advanced phase angle of entrainment and a shorter free-running period (Figure 4, A and B). After a prolonged follow-up of wheel-running behavior in LD (Figure 4C), we observed altered activity onset in Mfn2^–/–^ mice relative to the dark phase. Compared with the control animals, they had shifted activity onset toward the light phase, resulting in higher activity counts during the light phase and statistically lower activity counts during the dark phase, but without changes in their total activity level (Figure 4, D and E).

Next, when analyzing the core body temperature, we found a suggestively advanced phase shift with a decrease in average temperature values in Mfn2^–/–^ mice compared with control mice (Figure 4F). Furthermore, assessment of the sleep-wake pattern during the transition period from the light phase to the dark phase revealed that these mice slept 10%–15% less on average, even though we did not observe a significant difference between the 2 groups (Figure 4G). Conversely, the sleep-wake pattern during the transition from the dark to light phase was disrupted in Mfn2^–/–^ mice, with a significant increase in wakefulness at the expense of non-REM (NREM) sleep during the final hour (ZT23–ZT0) of the dark phase (Figure 4H). Collectively, the results suggest asynchronous circadian entrainment of bodily biological rhythms to the environmental LD cycle in mice with impaired mitochondrial dynamics in SCN VIP cells due to Mfn2 deletion.

## Discussion

The distinctly timed output from SCN VIP neurons is essential for the circadian synchronization of daily biological rhythms (21). However, cellular mechanisms and factors that regulate the activity of these neurons with a high degree of fidelity necessary for maintaining the homeostatic rhythmicity of an organism have not been fully elucidated. Here, we analyzed the crosstalk between mitochondria function in SCN VIP neurons and their circadian oscillatory output that governs biological rhythms of activity, body temperature, and sleep. We first observed the association of the mitochondrial fusion and fission dynamics with photic integration in these neurons. When this adaptive structural arrangement of SCN VIP mitochondria was disrupted by Mfn2 downregulation, we detected a whole spectrum of synaptic morphological and electrophysiological alterations evoking desynchronization of the SCN circuitry. These changes consequently resulted in circadian behavior dysrhythmia in Mfn2^–/–^ mice, with a significant impairment in their locomotor activity onset, core body temperature, and sleep-wake regulation.

It has recently been reported that developmental disruption of the molecular clock within SCN VIP neurons, or their genetic ablation in adult mice, leads to profound changes in wheel-running activity, body temperature, and sleep-wake rhythms (5). Interestingly, we showed that even less robust alteration in SCN VIP neurons, such as abnormality in their mitochondrial dynamics induced by Mfn2 loss of function was sufficient to trigger circadian dysrhythmia. This finding can be particularly important in the context of the daily rhythmicity changes commonly observed during aging and in age-related neurodegenerative diseases, given the evidence of a decline in mitochondrial functions therein (22, 23). These conditions are associated with impaired Mfn2-regulated mitochondrial fusion, which in turn affects the shape, distribution, and function of mitochondria as well as metabolic and bioenergetic properties of the cell (24, 25). In the SCN VIP neurons of Mfn2^–/–^ mice, we found abnormally enlarged, spherical mitochondria that lost their plasticity in daily morphological adaptations compared with control SCN VIP neurons. As a result, these Mfn2-deficient cells probably had a reduced ability to sustain proper endogenous respiration and efficient energy production, as demonstrated in previous studies (26, 27). In turn, this can adversely affect the VIP/VPAC2 neuropeptidergic signaling axis within the SCN, leading to functional disruptions in its downstream effector targets and circadian regulation.

In accordance, in Mfn2^–/–^ mice, we found impaired circadian locomotor behavior characterized by an advanced shift in activity onset compared with their control counterparts. This is consistent with the requisite role of SCN VIP neurons in regulating circadian locomotor activity, given their direct projections to the dorsomedial hypothalamic nucleus, which is shown to play an important role in this process (5, 28). Intriguingly, in contrast to the previously observed negative correlation between locomotor activity and SCN VIP activation (9), we did not detect any difference in total activity levels between the mutant and control mice, even though increased activation of these neurons in Mfn2^–/–^ animals was found using cFos labeling. This possibly indicates that in the condition of compromised functioning of VIP neurons, the other SCN cell populations may be involved in circadian activity regulation in a compensatory manner.

Considering that the local SCN output is intimately integrated with hypothalamic centers involved in thermoregulation and sleep-arousal states (28–30), we also monitored the LD fluctuations of body temperature and the sleep-wake pattern. In mice with Mfn2-depleted VIP neurons, we found a defective core body temperature rhythm, similar to that observed after total ablation of these neurons in the SCN (5). Specifically, we noticed a daily average body temperature reduction in these mice compared with controls during the 24-hour LD cycle. This result could reflect increased inhibitory input from SCN VIP neurons of Mfn2^–/–^ animals to the thermogenesis-promoting neurons in the dorsomedial hypothalamic nucleus-preoptic area circuit, which is implicated in body temperature regulation based on ambient temperatures (31, 32). When testing whether disruption of mitochondrial dynamics in SCN VIP neurons affected sleep-wake patterns during LD and DL transition periods, we found that Mfn2^–/–^ mice exhibited markedly increased wakefulness during the last hour of the dark phase in the DL transition compared with control mice. This likely resulted from increased activation of SCN VIP neurons that we observed in these animals during the dark phase and conceptually aligns with previous findings of heightened total SCN activity during the waking episodes (33). Although measured in the restricted time window, i.e., only during phase transitions, this finding suggests altered sleep-wake behavior in Mfn2^–/–^ mice. The role of SCN VIP neurons in circadian sleep-wake regulation is ambiguous, given the evidence showing that their activation does not directly modulate sleep and wake length and distribution across the day and night (5), as well as those reporting the essential role of the activation of these neurons in sculpting the nighttime but not the daytime sleep-wake rhythm (29). Our findings align with the latter study regarding the involvement of these neurons in circadian gating of sleep, but given that sleep lies under the control of circadian as well as homeostatic regulation (34), it remains plausible that altered SCN activity could affect homeostatic sleep drive per se. Yet, given the restricted time window of our recordings here, it remains challenging to disentangle how exactly SCN VIP neurons regulate sleep/wake behavior, underscoring the need for further study. In a broader view, these results for sleep-wake pattern changes in Mfn2^–/–^ mice also provide new data for the emerging role of mitochondria in sleep control (35), which has not been extensively studied thus far, in contrast to ample existing evidence showing how sleep affects mitochondrial function and fitness (36).

Taken together, our data indicate the importance of proper mitochondrial dynamics in SCN VIP neuronal output as a critical effector mechanism for shaping the biological rhythms of a mammalian organism in response to the circadian daily cycle.

## Methods

### Sex as a biological variable

Our study examined male and female animals, and similar findings are reported for both sexes in all experiments.

### Mice

All mice were maintained in temperature- and humidity-controlled rooms under a 12-hour light/12-hour dark cycle (lights on at 0700 hours, lights off at 1900 hours) unless otherwise stated. Food and water were provided ad libitum. Both VIP-IRES-Cre mice (Vip*^tm1(cre)Zjh^*/AreckJ; strain JAX:031628) and their background control C57BL/6J mice (strain JAX:000664) were purchased from The Jackson Laboratory. The VIP-IRES-Cre mice have Cre recombinase expression directed to Vip-expressing cells by the endogenous promoter/enhancer elements of the vasoactive intestinal polypeptide locus (*Vip*) on chromosome 10. To generate conditional Mfn2^–/–^ mice in VIP neurons, the mice were crossed with Mfn2-floxed mice (Mfn2*^tm3Dcc^*/Mmucd; strain MMRRC_029902-UCD) available in our laboratory using the previously described approach (37, 38). To visualize VIP neurons, Cre-dependent tdTomato reporter control Ai14 mice (Gt(ROSA)26Sor*^tm14(CAG-tdTomato)Hze^*/J; strain JAX:007914) crossed with Mfn2^–/–^ mice were used for histological analysis and ex vivo electrophysiological recordings at the age of 1.5–3 months. Mice between 2–6 months of age were used for in vivo electrophysiological recordings, as well as body temperature and wheel-running measurements.

### Immunostaining

Mice were anesthetized and transcardially perfused with ice-cold saline (0.9% NaCl) containing heparin (10 mg/L), followed by a fixative solution (4% paraformaldehyde in 0.1 M phosphate buffer [PB], pH 7.4). Brains were postfixed overnight at 4°C. Vibratome-cut 50 μm thick sections were washed in PB for 15 minutes and then incubated in blocking solution (5% normal donkey serum in PB) with 0.3% Trition X-100 for 1 hour at room temperature. Sections were incubated with the primary antibodies rabbit anti-VPAC2 (1:300; catalog ab183334, Abcam) and goat anti-cFos (1:1,000; catalog sc-52-G, Santa Cruz Biotechnology) for 48 hours at 4°C, and then washed 3 times in PB for 15 minutes each at room temperature, followed by incubation with the respective secondary antibodies donkey anti–rabbit 647 (1:700, catalog A31573, Thermo Fisher Scientific) and donkey anti–goat 488 (1:700, catalog A11055, Thermo Fisher Scientific) overnight at 4°C. For VIP neuron labeling, sections were incubated with rabbit anti-VIP antibody (1:500; catalog 20077, ImmunoStar) for 72 hours at 4°C and then with donkey anti–rabbit 647 (1:500; catalog A31573, Thermo Fisher Scientific) for 2 hours at room temperature. The following day, the sections were washed and coverslipped using Vectashield mounting medium (H-1000, Vector Laboratories). All analyses were done using a Leica Stellaris 5WLL microscope (Leica Microsystems).

### Electron microscopy

Mice were anesthetized and first perfused with ice-cold saline (0.9% NaCl) containing heparin (10 mg/L) followed by a fixative solution (4% paraformaldehyde, 15% vol/vol picric acid, 0.1 vol/vol glutaraldehyde in 0.1 M PB). Brains were postfixed in a fixative solution without glutaraldehyde overnight at 4°C. For VIP cell visualization, 50 μm thick brain tissue sections that contained the SCN were first processed in 10% and 20% sucrose solution for 30 minutes each, and after a freeze-thaw step, they were incubated with rabbit anti-VIP antibody (1:1,000; catalog 20077, ImmunoStar) for 72 hours at 4°C. The next day, sections were washed, incubated with goat anti–rabbit HRP–conjugated antibody (1:200; catalog 12-348, MilliporeSigma) for 1.5 hours, washed again, and developed with DAB. Subsequently, sections were osmicated and dehydrated in ethanol. Ultrathin sections cut using a Leica Ultra-Microtome were collected on Formvar-coated, single-slot grids and analyzed with a Tecnai 12 Biotwin electron microscope (TEM-FEI).

### Image analysis

The mitochondrial cross-sectional area, perimeter, circularity, and aspect ratio were calculated using the “analyze particles” function in ImageJ (NIH) (https://imagej.net/imaging/particle-analysis). Mitochondrial density was calculated by dividing the number of mitochondria in the cytosolic area. Mitochondrial coverage was calculated by dividing the total area of mitochondria by the cytosolic area. For synapse density, an investigator blinded to the experimental protocol measured the number of synapses, as previously described (39). For immunofluorescence image analyses, *Z*-stack images of brain sections were collected to manually count the cFos-expressing cells by an observer blinded to the experimental conditions. The measurement of the immunofluorescence intensity of VIP in SCNs was calculated using ImageJ. The background intensity of the SCN area was used to normalize the raw integrated density (RawIntDen) of the VIP antibody. The average value of 2 sides of SCNs was used per section.

### Electrophysiology

#### Sleep recording.

Sleep recording was carried out in freely behaving male mice implanted with chronically indwelling electrodes. After achieving a surgical plane of isoflurane anesthesia (induction 4%, maintenance 1.5% in O_2_), mice were placed in Kopf stereotaxic frame, and stainless screw electrodes (Plastics One) were implanted under aseptic conditions over the right frontal and parietal cortices for electroencephalographic (EEG) recordings and a wire electrode with suture pad inserted deep into the neck muscle tissue for monitoring electromyographic (EMG) activity. To serve as a reference, 2 additional screws were placed over the cerebellum. All electrodes were joined to a miniature connector, which was affixed to the skull using dental acrylic, and the skin incision was closed with nylon sutures. After surgery, each mouse was kept in a clean cage, and all necessary postoperative care was taken. Following a 10-day recovery period, mice in their individual home cages were connected to slip-ring commutators with flexible cables and left undisturbed for habituation of at least 24 hours before EEG/EMG recordings. All recordings were done during the transition periods during the LD cycle, i.e., during the last hour of light or dark (ZT11–12 or ZT23–0) and the first 2 hours after shifting in the opposite phase (ZT12–14 or ZT0–2). The light intensity in the procedure room during recordings was approximately 350 lux with the light on and around 0.1 lux with the light off. The acquired EEG/EMG signal was filtered between 1 and 500 Hz using the A-M System (model 1800) with an additional notch filter at 60 Hz, simultaneously digitized at a rate of 1 kHz, and stored on a computer via CED Micro1401-3 interface and Spike2, version 9, software (Cambridge Electronic Design). Subsequent analyses were performed offline and by operators blinded to genotype in consecutive 5-second-long epochs using Spike2 scripts for automatic sleep staging and scoring according to standard criteria for rodents as described previously (40). Epochs scored as ambiguous (approximately <5%), occurring mainly due to occasional artifacts in the EEG signal, were excluded from the analysis.

#### Ex vivo SCN recording.

Control and mutant Mfn2^–/–^ male mice were deeply anesthetized with isoflurane and decapitated at ZT7. Their brains were then rapidly removed, and coronal tissue sections of 300 μm thickness at the rostro-caudal level of SCN (41) were made using a vibratome in an oxygenated (5% CO_2_ plus 95% O_2_) cutting solution at 4°C that contained 220 mM sucrose, 2.5 mM KCl, 1 mM CaCl_2_, 6 mM MgCl_2_, 1.25 mM NaH_2_PO_4_, 26 mM NaHCO_3_, and 10 mM glucose, pH 7.3, with NaOH. After preparation, the slices were transferred to a recording chamber constantly perfused at a rate of 2 mL/min with artificial cerebrospinal fluid (containing 124 mM NaCl, 3 mM KCl, 2 mM CaCl_2_, 2 mM MgCl_2_, 1.23 mM NaH_2_PO_4_, 26 mM NaHCO_3_, 10 mM glucose, pH 7.4, with NaOH) at 33°C.

Whole-cell voltage-clamping (at –60 mV or 0 mV) was performed to measure miniature excitatory and inhibitory postsynaptic currents (mEPSC and mIPSC) using a Multiclamp 700 A Axon Instruments amplifier (Molecular Devices). The patch pipettes with a tip resistance of 4–6 MΩ were made of borosilicate glass (World Precision Instruments) with a Sutter pipette puller (P-97) and filled with a pipette solution containing 135 mM K-gluconate, 2 mM MgCl_2_, 10 mM HEPES, 1.1 mM EGTA, 2 mM Mg-ATP, 10 mM Na_2_-phosphocreatine, and 0.3 mM Na_2_-GTP, pH 7.3, with KOH. After the giga-ohm (GΩ) seal and whole-cell access were achieved, the series resistance (10–20 MΩ) was partially compensated by the amplifier. All data were sampled at 10 kHz, filtered at 3 kHz, analyzed with the AxoGraph X event detection package (AxoGraph), and plotted with Igor Pro software (WaveMetrics).

Multiunit activity (MUA) in the SCN was recorded following slice preparation as above using a 16-channel silicone microelectrode linear array with a 50 μm shank separation (A16x1 NeuroNexus) attached to the micromanipulator. The signals were amplified with an A-M System amplifier (model 3600), sampled at 10 kHz, and stored on the computer disc using the Micro1401-3 and Spike2 (Cambridge Electronic Design [CED]). For every mouse, 6 recording sites spanning the entire dorsoventral dimension of the SCN were identified from pictures taken at the end of each recording and subsequently analyzed with Spike2 software. To test SCN activity synchronization, instantaneous population rates were extracted for the dorsal and ventral neuronal populations using 0.5-second moving windows with 50% overlap, and the cross-correlation between the 2 was computed.

### Body temperature measurement

Mice were injected with buprenorphine (Ethiqa XR, 3.25 mg/kg, subcutaneously) and anesthetized with isoflurane. The abdominal skin was then shaved, and a 1.5 cm incision was made at the abdominal midline. Real-time readable temperature telemetry transmitters (Anilogger system, Bodycap) were implanted into the intraperitoneal cavity ventral to the caudal arteries and veins but dorsal to the abdominal viscera. Incisions were sutured and treated with a topical antibiotic, and each animal was placed in an individual cage and received subcutaneous injections of analgesic (meloxicam, 5 mg/kg) for 2 days. Following a 10-day recovery at an ambient temperature (22°C ± 1°C) and on a 12-hour light/12-hour dark cycle with food and water available ad libitum, the core body temperatures of the mice were measured every 15 minutes over a 24-hour period. Data were acquired using the portable telemetry receiver, transferred to a computer as an Excel file, and analyzed using GraphPad Prism (GraphPad Software).

### Wheel-running behavior

Mice were placed in individual wheel-running cages and allowed free access to food and water. Locomotor activity was recorded using the ClockLab Data Collection System (Actimetrics). Activity data was analyzed in 6-minute bouts using ClockLab software (Actimetrics). The free-running period was determined by line-fitting of activity onsets from data collected during the DD period.

### Statistics

Statistical analyses were performed using an unpaired, 2-sided *t* test, a Mann-Whitney *U* test, a 1- or 2-way ANOVA with Tukey’s post hoc test, and a Kruskal-Wallis tests with Dunn’s post hoc test in GraphPad Prism 10 (GraphPad Software). Normality was assessed using the Shapiro-Wilk test, and the choice of parametric or nonparametric tests was based on the results, as detailed in Supplemental Table 1. Cumulative distributions of mEPSC and mIPSC measurements were compared using the Kolmogorov-Smirnov test. For temperature variation analysis, a nonlinear regression fit was applied using a sine function constrained to a 24-hour wavelength. A *P* value of less than 0.05 was considered significant. All data are presented as the mean ± SEM. The number of replicates for each experiment is specified in the figure legends.

### Study approval

All mouse studies were designed and performed in accordance with NIH guidelines and animal protocols approved by the IACUCs of Yale University and Northwestern University.

### Data availability

The data used to support the findings of this study are available within this article, supplemental material, and the Supporting Data Values file. Recordings of sleep, multiunit activity, and wheel-running behavior have been deposited in the GIN open data management system (https://gin.g-node.org/Horvath_Lab/Mfn2-VIP-SCN.git).

## Author contributions

MS, JES, HKH, HE, LV, J Catarino, XBG, ZWL, J Cedernaes, JB, and TLH conducted the experiments and analyzed data. SD and PS analyzed data. MS, JES, and TLH designed the study and wrote the paper with input from all authors.

## Supplementary Material

Supplemental data

Supporting data values

## Version 1 07/01/2025

Electronic publication

## Version 2 07/11/2025

Name of Joseph Bass corrected in author list.
