# Supplementary material for: Mitofusin 2 controls mitochondrial and synaptic dynamics of suprachiasmatic VIP neurons and related circadian rhythms
Source: J Clin Invest. 2025 Jul 1;135(13):e185000. doi: 10.1172/JCI185000 (PMC12208536; doi:10.1172/JCI185000)
Supplement: Supplemental data [file jci-135-185000-s005.pdf]

Supplemental material

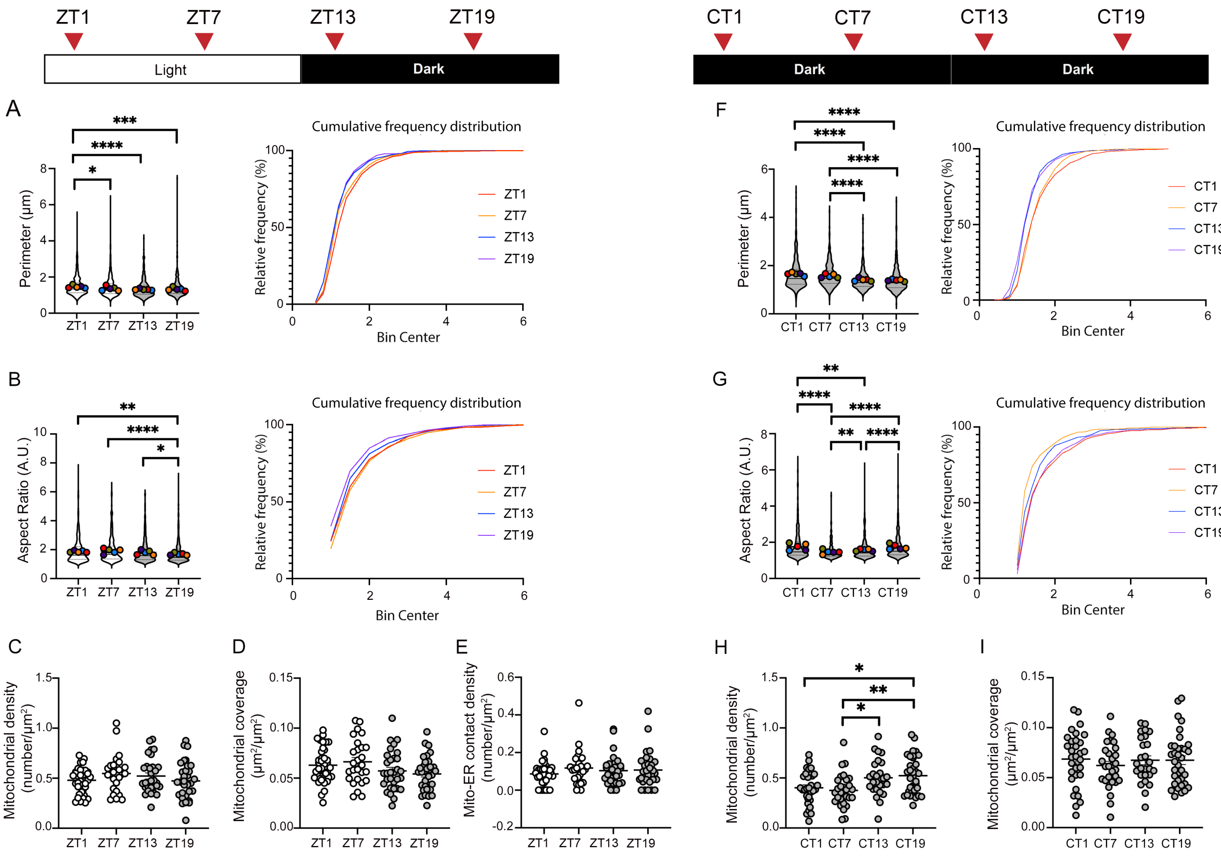

Figure S1. Mitochondrial morphology of SCN VIP neurons in light-dark (LD) and constant darkness (DD) environment.

(**A-E**) Electron microscopy image analyses of SCN VIP-immunolabeled neurons from C57BL/6J mice housed in LD condition (ZT0: light on; ZT12: light off). (**A**), Cross sectional perimeter; (**B**), Aspect ratio of mitochondria in SCN VIP neurons and their cumulative probability distributions at ZT1, ZT7, ZT13, ZT19 in LD. (**C**), Mitochondrial density; (**D**), Mitochondrial cytosol coverage; (**E**), Mitochondria-ER contact per SCN VIP neuron at ZT1, ZT7, ZT13, ZT19 in LD. (**F-I**) Electron microscopy image analyses of SCN VIP-immunolabeled neurons from C57BL/6J mice housed in DD condition for 48 hours. (**F**), Cross sectional perimeter; (**G**), Aspect ratio of mitochondria in SCN VIP neurons and their cumulative probability distributions at CT1, CT7, CT13, CT19 in DD. (**H**), Mitochondrial density; (**I**), Mitochondrial cytosol coverage per SCN VIP neuron at CT1, CT7, CT13, CT19 in DD. Approximately 5 cells per mice; 5 mice per time point. Supplemental Table S1 lists statistical information for each graph. *P<0.05; **P<0.01; ***P<0.005; ****P<0.0001.

Table S1. Statistics underlying Figures and Supplemental Figure

Figure	Data structure	Type of test	Test result
1A total	heterogenous distribution	Kruskal-Wallis with Dunn's test	H = 14.17, P=0.003
1A excit	heterogenous distribution	Kruskal-Wallis with Dunn's test	H = 15.90, P=0.001
1A inhib	heterogenous distribution	Kruskal-Wallis with Dunn's test	H = 6.83, P=0.078
1C	heterogenous distribution	Kruskal-Wallis with Dunn's test	H = 39.24, P<0.001
1D	heterogenous distribution	Kruskal-Wallis with Dunn's test	H = 30.51, P<0.001
1F	normal distribution	2-way ANOVA with Tukey's test	$F_{(15, 60)}$ = 55.10, P<0.001
1G total	heterogenous distribution	Kruskal-Wallis with Dunn's test	H = 3.59, P=0.309
1G excit	heterogenous distribution	Kruskal-Wallis with Dunn's test	H = 5.06, P=0.167
1G inhib	heterogenous distribution	Kruskal-Wallis with Dunn's test	H = 1.24, P=0.743
1H	heterogenous distribution	Kruskal-Wallis with Dunn's test	H = 129.5, P<0.001
1I	heterogenous distribution	Kruskal-Wallis with Dunn's test	H = 89.59, P<0.001
1J	normal distribution	2-way ANOVA with Tukey's test	$F_{(15, 72)}$ = 27.85, P<0.001
2C	heterogenous distribution	Kruskal-Wallis with Dunn's test	H = 172.7, P<0.001
2D	normal distribution	1-way ANOVA with Tukey's test	$F_{(3, 101)}$ = 3.10, P=0.03
2E	heterogenous distribution	Kruskal-Wallis with Dunn's test	H = 14.07, P=0.002
2G	heterogenous distribution	Kruskal-Wallis with Dunn's test	H = 44.88, P<0.001
2H	heterogenous distribution	Kruskal-Wallis with Dunn's test	H = 131.7, P<0.001
2I	heterogenous distribution	Mann Whitney test	U = 394, P=0.311
2J total	heterogenous distribution	Kruskal-Wallis with Dunn's test	H = 1.002, P=0.801
2J excit	heterogenous distribution	Kruskal-Wallis with Dunn's test	H = 7.47, P=0.058
2J inhib	heterogenous distribution	Kruskal-Wallis with Dunn's test	H = 3.87, P=0.275
2K amp	heterogenous distribution	Kolmogorov-Smirnov test	D = 0.091, P=0.014
2K freq	heterogenous distribution	Mann Whitney test	U = 80, P=0.01
2L amp	heterogenous distribution	Kolmogorov-Smirnov test	D = 0.160, P<0.001
2L freq	heterogenous distribution	Mann Whitney test	U = 118, P=0.043
3C	heterogenous distribution	Kruskal-Wallis with Dunn's test	H = 25.89, P<0.001
3D	normal distribution	1-way ANOVA with Tukey's test	$F_{(3, 51)}$ = 3.86, P=0.014
3E	normal distribution	1-way ANOVA with Tukey's test	$F_{(3, 53)}$ = 126.2, P<0.001
3F	normal distribution	1-way ANOVA with Tukey's test	$F_{(3, 55)}$ = 95.08, P<0.001
3G	normal distribution	unpaired t test	t = 2.23, df=34, P=0.032
3H	heterogenous distribution	Mann Whitney test	U = 0, P<0.001
3H corr.	normal distribution	unpaired t test	t = 2.20, df=4, P=0.046
4B	normal distribution	unpaired t test	t = 8.20, df=32, P<0.001
4D	normal distribution	unpaired t test	t = 5.08, df=32, P<0.001
4E	normal distribution	1-way ANOVA with Tukey's test	$F_{(5, 96)}$ = 51.48, P<0.001
4F temp	heterogenous distribution	Mann Whitney test	U = 2256, P<0.001
4F phase	normal distribution	unpaired t test	t = 2.02, P=0.089
4G	normal distribution	multiple unpaired t tests with Welch corrections	ZT11-12:P=0.22(W); P=0.47(NR);P=0.39(R); ZT12-14:P=0.21(W); P=0.25(NR);P=0.18(R)
4H	normal distribution	multiple unpaired t tests with Welch corrections	ZT23-0:P=0.02(W);P=0.014(NR);P=0.154(R); ZT0-2:P=0.656(W); P=0.553(NR);P=0.449(R)
S1A	heterogenous distribution	Kruskal-Wallis with Dunn's test	H = 28.62, P<0.001
S1B	heterogenous distribution	Kruskal-Wallis with Dunn's test	H = 25.21, P<0.001
S1C	heterogenous distribution	Kruskal-Wallis with Dunn's test	H = 3.29, P=0.349
S1D	normal distribution	1-way ANOVA with Tukey's test	$F_{(3, 125)}$ = 2.55, P=0.005
S1E	heterogenous distribution	Kruskal-Wallis with Dunn's test	H = 2.05, P=0.561
S1F	heterogenous distribution	Kruskal-Wallis with Dunn's test	H = 94.29, P<0.001
S1G	heterogenous distribution	Kruskal-Wallis with Dunn's test	H = 73.02, P<0.001
S1H	normal distribution	1-way ANOVA with Tukey's test	H = 1.28, P=0.732
S1I	heterogenous distribution	Kruskal-Wallis with Dunn's test	$F_{(3, 117)}$ = 5.39, P=0.001

Shapiro-Wilk test was used for assessing data normality and lognormality.
